# Developing a novel risk-scoring system for predicting relapse in patients with ulcerative colitis: A prospective cohort study

**DOI:** 10.12669/pjms.316.8811

**Published:** 2015

**Authors:** Seyed Vahid Hosseini, Ali Reza Safarpour, Seyed Alireza Taghavi

**Affiliations:** 1 Seyed Vahid Hosseini, Colorectal Research Center, Faghihi Hospital, Shiraz University of Medical Sciences, Shiraz, Iran; 2 Ali Reza Safarpour, Gastroenterohepatology Research Center, Namazi Hospital, Shiraz University of Medical Sciences, Shiraz, Iran; 3 Seyed Alireza Taghavi, Gastroenterohepatology Research Center, Namazi Hospital, Shiraz University of Medical Sciences, Shiraz, Iran

**Keywords:** Ulcerative Colitis, Relapse, Cohort Study, Prediction

## Abstract

**Objectives:**

Ulcerative Colitis (UC) follows a natural clinical course of relapses and remissions. The aim of this study was to construct a risk-scoring formula in order to enable predicting relapses in patients with UC.

**Methods:**

From October 2012 to October 2013, 157 patients from Shiraz, southern Iran who were diagnosed with UC and in remission were enrolled. At 3-month intervals, multiple risk factors of hemoglobin, complete blood counts, serum iron and albumin, erythrocyte sedimentation rate, and faecal calprotectin levels, sex, age, cigarette smoking, positive family history of inflammatory bowel diseases, past history of appendectomy, extra-intestinal accompanying diseases, extent of disease at the beginning of study, number of previous relapses, duration of disease and duration of remission before the study were assessed. Univariate and multivariate logistic regression were applied to fit the final model. The new risk-scoring system accuracy was assessed using receiver-operating-characteristics (ROC) curve analysis.

**Results:**

Seventy four patients (48.1%) experienced a relapse. Multivariate analysis revealed that relapses could significantly be predicted by the level of fecal calprotectin (OR=8.1), age (OR=9.2), the Seo activity index (OR=52.7), and the number of previous relapses (OR=4.2). The risk scoring formula was developed using the regression coefficient values of the aforementioned variables.

**Conclusion:**

Four predictor variables were significant in the final model and were used in our risk-scoring formula. It is recommended that patients who achieve high scores are diligently observed, treated, and followed up.

## INTRODUCTION

Inflammatory bowel diseases (IBD), Crohn’s disease (CD) and ulcerative coloitis (UC), are the major gastrointestinal pathology, which have affected millions of people around the world. Increased incidence of the diseases in the developing countries is demonstrated in recent reports.^1^ The course of ulcerative colitis (UC) varies from decreased intestinal manifestations to extremely severe systemic signs and symptoms.^2^ Prevalence of the recurrence of intestinal and systemic presentations in patients with UC is relatively high. In a recent study, the risk of the first relapse after one to five years was 51% and 75%, respectively.^3^

Identifying patients at high risk of relapse and early onset of severe treatment (necessitating the use of early aggressive treatment) can prevent serious complications of the disease.^4^ Obviously, the initiation of aggressive treatment in patients who do not need such treatment could lead to complications such as severe infections^5^ or even carcinoma.^6^ Therefore, in order to prevent relapse and grave complications in patients with anticipated relapse it is critical to use clinical, demographic, or histological predictive factors.^7^

Patients that are recognised to be at higher risk of disease exacerbation could be candidates for appropriate treatment and better monitoring.^8^ The goal of the present study was to calculate a simple and yet accurate risk-score to help prevent relapses in patients with UC.

## METHODS

### Study design, settings, and sample size

In a prospective cohort study, patients with UC, who were at least three months in clinical remission were included. The patients were from the Gastroenterohepatology Research Center Registry in Shiraz, Fars Province, southern Iran (population 4,597,000).^9^,^10^ According to previous studies, the sample size was calculated as 135 patients which increased to 157 due to probable lost to follow up.

The study’s sampling frame comprised all registered patients (1,273 ulcerative colitis patients) in the registry. Systematic random sampling was done to select a calculated sample size with a sampling interval of 8 (1,273/157). The follow-up was over one year period from 1st October 2012 to 1st October 2013. The patients were followed up at interval of three months. All 157 patients were followed to the end of the study unless they had a relapse. Two patients did not complete the study. In addition, one patient was excluded from the study because of relapse due to arbitrary cut-off drugs.

### Participants and case definition

Patients with confirmed diagnosis of UC, according to the Truelove and Witt’s criteria, were included.^11^ Clinical relapse was described as the deterioration of bowel movements (accompanied by lower intestinal bleeding) or worsening of the abdominal pain and diarrhoea, leading to change in previous treatment (increase in dose or change of drugs), added steroids, admission to hospital or surgery and high (>220) Seo index.^12^,^13^

Clinical remission was characterized by normal baseline stool frequency and the lack of bloody stool and the pathology report of no active inflammatory activity in three months prior to the study.^12^ Pregnant patients were also excluded.

### Follow-up and data collection

The patients who developed relapse during the study were excluded from further follow-up. At the first and each subsequent follow-up, all clinical laboratory assessments and disease-related data were collected using a questionnaire designed by an expert trained general practitioner. During the visits, UC severity forms were also completed according to Seo index criteria. The Seo index was calculated using the following formula and recorded at 0, 3, 6, 9 and 12 months, or at the time of relapse:

Seo index=(60×BS per day)+(13×BM per day)+(0.5×ESR)-(4×Hb)-(15×Alb)+200

Where BS is bloody stool and BM is bowel movements.^14^,^15^

### Variables and outcome

The primary outcome was the occurrence of relapse within 12-month follow-up period. During the follow up, blood and stool samples were collected from the patients quarterly. The samples were tested for complete blood counts, serum iron, serum albumin, erythrocyte sedimentation rate, and faecal calprotectin (FC) levels. Disease-related and demographic variables were recorded at the start of the study.

### Ethics

The Shiraz University of Medical Sciences Ethics Committee evaluated and approved the study (code 6495). All patients read the study objectives (or debriefed verbally) and written informed consent was obtained.

### Laboratory investigations

General laboratory assays, e.g., WBC count, hemoglobin (Hb), erythrocyte sedimentation rate (ESR) and platelet count were performed according to standard laboratory procedures. The chemical and kinetic colorimetric methods were used to determine serum iron concentration and serum albumin (Alb) levels, respectively. A commercially accessible enzyme-linked immunosorbent assay (ELISA) was used to quantitatively determine the fecal calprotectin (FC) levels (Buglmann Laboratories AG, Schonenbuch, Switzerland).^16^

### Statistical analysis

Univariate logistic regression analysis was used to differentiate potential variables that have an effect on the occurrence of relapse. To control confounders and find the variables related to relapse that fit the final predictive model, a multiple logistic regression analysis was performed. Forward stepwise likelihood ratio method was taken in the selection of final variables in the model. P values less than 0.05 were considered significant. The SPSS statistical software, version 16 (Inc. Chicago, IL) was used to carry out data analyses.

### Development of risk-score

Our method for the development of a new risk-score for the prediction of relapse was very similar to the method previously used by Ho et al.^17^ Due to the wide range of regression coefficients for each variable in the regression model, ROC curve analysis was used to change quantitative variables to binary variables (according to cut-off points). Maximum sensitivity and specificity with strong clinical relevance and Youden’s index were the selection criteria of cut-off points.

Initially, we determined the absolute values of regression coefficients. The positive decimal numbers were then rounded to integers. If β_1…_β_p_ were the regression coefficients of dichotomous independent variables, including X_1_, X_2_, …, X_P_, then the score was developed using the following formula:

Risk Score=RND|β_1_|×X_1_+RND|β_2_|×X_2_+RND|β_3_|×…+ X_p_ ×RND|β_p_|

Where RND is a rounding function, which returns decimal numbers to integers and |β_i_| is the absolute value of the regression coefficients in the final model. In this formula, X_i_ takes value 1 if the patient is at risk for relapse.

## RESULTS

During the one-year follow-up period 154 patients continued the study until its completion. 48.7% were women with a mean age of 42.48±11.22 years and a range of 20-69 years. Men represented the remaining 51.3% of the study with the mean age of 41.81±10.82 and a range of 21-83 years. Mean follow up time was 232 days. Relapse rate were 41.8% and 54.7% in male and female, respectively, (*P*=0.14).

### Univariate analysis of independent variables

Uivariate comparison of the variables, revealed that age, pre-study disease and remission durations, Hb, serum Alb, Seo index, ESR rate, white blood cells, FC levels, and cigarette smoking had statistically significant differences between the relapsers and non relapsers (*P*< 0.1). Table-I.

**Table-I:** Baseline variables in relapsers and non-relapsers with univariate analysis of relapse (n=154).

P- Value	Odds Ratio (95%) CI	Non- Relapsers	Relapsers	Overall	Variables
Age(mean,SD)	42.13(10.1)	36.90 (8.9)	46.9(10.56)	0.89(0.85-0.93)	< 0.001
*Sex*					
Male	79(51.3%)	33(41.8%)	46(58.2%)	1.68(0.88-3.18)	0.1
Female	75(48.7)	41(54.7%)	34(45.3%)		
Duration of disease before study (month) (mean,SD)	99.8(62.3)	88.9(57.9)	109.88(64.82)	0.99(0.98-1.00)	0.036
Duration of remission before study (month) (mean,SD)	46.55(40.61)	37.39(32.36)	55.02(45.36)	0.98(0.98-0.99)	0.007
Number of previous relapses (mean,SD)	3.57(3)	5.02(3.29)	2.22(1.90)	1.55(1.32-1.82)	<0.001
Seo index	194.96(129.17)	278.07(142.32)	118.07(33.46)	1.36(1.02-1.04)	<0.001
*Laboratory Variables(mean,SD)*					
ESR(mm/h)	17.01(11.71)	21.09(13.30)	13.23(8.49)	1.07(1.03-1.11)	<0.001
Alb(g/L)	4.41(0.45)	4.16(0.48)	4.64(0.25)	0.02(0.008-0.08)	<0.001
WBC(10^9^/L)	6.29(1.74)	7.28(1.78)	5.38(1.10)	1.00(1.0-1.01)	<0.001
Platelets(10^9^/L)	247.43(145.5)	268.43(20.2)	228.58(49.7)	1.0(1.0-1.0)	0.09
FC(μg/g)	499.33(593.63)	862.82(655.97)	163.19(215.83)	1.05(1.01-1.07)	<0.001
Iron(μg/g)	13.09(1.33)	13.09(1.33)	13.11(1.36)	0.96(0.76-1.2)	0.794
Hb (g/L)	13.82(1.980	13.36(1.77)	14.25(2.08)	0.77(0.6-0.9)	0.005
*Cigarette smoking*					
Non-smokers	119(77.3%)	51(68.9%)	68(85%)	2.55(1.16-5.61)	0.01
Current-smokers	35(22.7%)	23(31.1%)	12(15%)		
*Appendectomy*					
Yes	5(3.2%)	4(5.4%)	1(1.2%)	0.22(0.024-.002)	0.14
No	149(96.8%)	70(94.6%0	79(98.8%)		
*Extra intestinal manifestations*					
Yes	65(42.2%)	29(39.2%)	36(45%)	1.27(0.66-2.41)	0.46
No	89(57.8%)	45(60.80%)	44(55%)		
*Positive family history*					
Yes	18(11.7%)	8(10.8%)	10(12.5%)	1.17(0.43-3.14)	0.74
No	136(88.3%)	66(89.2%)	70(87.5%)		
*Extent of colitis on diagnosis*					
Proctitis	83(53.9)	36(48.6%)	47(58.8%)	1.50(0.79-2.84)	0.20
Left sided/Pan colitis	71(46.1)	38(51.4%)	33(41.2%)		

### Multiple logistic regression analysis and final model

Multiple logistic regression analysis demonstrated four final independent variables: FC, number of previous relapses (NPR), age, and Seo index were significantly associated with relapse. According to ROC curve analysis FC ≥ 341µg/g, NPR ≥2 times, age ≤42.5 years and Seo index ≥148.3, contributed to the highest risk of relapse. For easier interpretation absolute and rounded numbers of regression coefficients of the FC, NPR, age and Seo index (2, 2,1and 4, respectively) were used to generate a new risk-scoring formula. (Table-II). In the present study, Hosmer-leme show statistic as a measure of the goodness of fit of the final model was used. The value of that was 5.69 (P=0.58). Therefore we concluded that the fitted model is satisfactory.

**Table-II:** Multiple logistic regression analysis for previous significant variables.

Independent variables	Coefficient	Odds ratio	95% CI		P-value
			Lower	Upper	
FC*					
≥ 341					
< 341	2.096	8.13	2.33	28.35	0.001
NPR**					
≥ 2					
< 2	1.44	4.22	1.21	14.75	0.024
Age(at diagnosis)					
≤ 42.5					
> 42.5	2.22	9.24	2.29	37.24	0.002
Seo index					
≥ 148.3					
< 148.3	3.96	52.77	11.86	234.76	<.001
Duration of remission before study	-	-	-	-	0.11
Duration of disease before study	-	-	-	-	0.38
White blood cells	-	-	-	-	0.56
Cigarette smoking	-	-	-	-	0.25

* Fecal calprotectin,

** Number of previous relapses.

### According to the method explained in the previous section, the following formula was obtained

Risk Score= (4×Seo index)+(2×NPR)+(2×FC)+(1×age)

**Fig.1:**
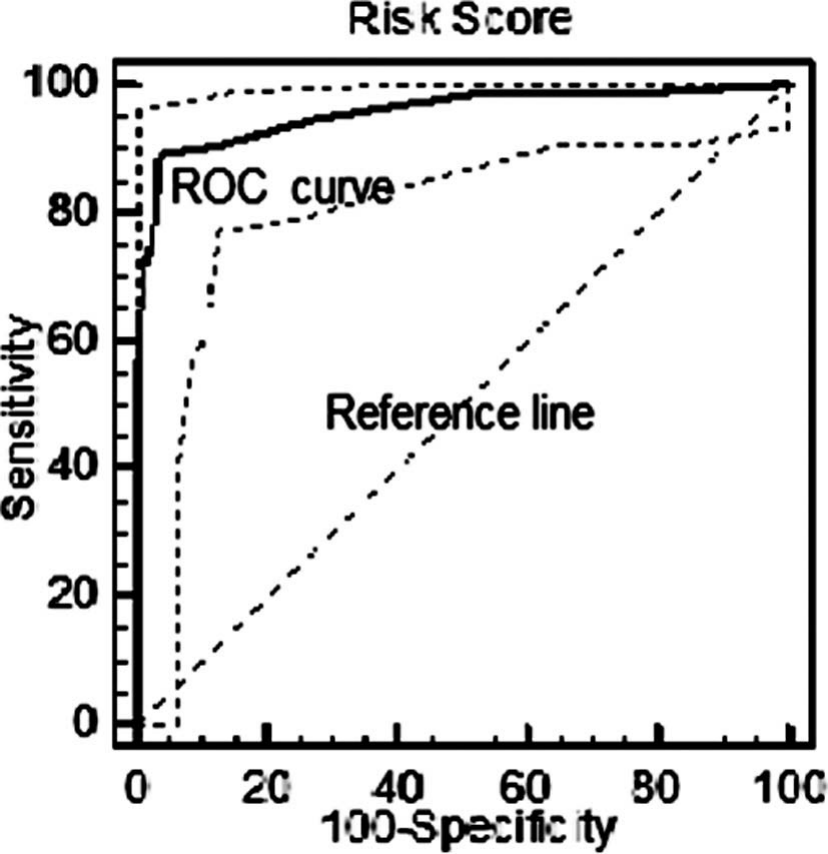
ROC curve demonstrated that final fitted model is adequate.

This formula uses the coefficients of the regression analysis reported in Table-II. The faecal calprotectin (FC) ≥341, number of previous relapses (NPR) ≥2, age at diagnosis ≤42.5, and Seo index ≥148.3 were given the scores of 2, 2, 1, and 4, respectively. According to the above formula, the risk-score for a single patient is in the range of 0 to 9. The minimum score (zero) would be attributed to a patient with FC <341, NPR <2.5, age >42.5, and Seo index <148.3, having the lowest risk of relapse. Discriminate validity for final risk score was assessed (P<0.001). ROC curve analysis was used for the evaluation of sensitivity and specificity of the scores for all relapsing and non-relapsing patients. It, revealed that patients with scores ≥6.5 had a high risk of relapse (sensitivity=80%, specificity=97.1%, and AUC=0.957) (Fig.1).

**Table:** 

Area under the ROC curve (AUC)	0.957
Significance level P (area=0.5)	<0.0001
Youden index	0.85
Sensitivity	80%
Specificity	97.1%

## DISCUSSION

This cohort study, has proposed a new way of risk-score for predicting relapse in patients with ulcerative colitis. Four variables, FC, NPR, age, and Seo index were finally included in the formula and it revealed significant predictive ability for prediction of relapse.

To the best of our knowledge, the present study is the first one that has been conducted on the accuracy of the Seo index in predicting relapse in UC. The very strong role of this variable in the prediction of relapse in our study may be due to the cumulative effect of Seo index fundamental variables (such as Hb, ESR and serum albumin).

The sensitivity and specificity of calprotectin in predicting relapse in patients with ulcerative colitis have been estimated respectively at 90% and 83%.^18^ Costa et al. showed that increased levels of calprotectin in stools had positive and negative predictive values of 81% and 90%, respectively.^19^ There have been many studies as regards to the limit of stool calprotectin, but there is an increased probability of relapse in patients with ulcerative colitis.

In Osterman’s study, clinical relapse in patients with FC ≥200 μg/g occurred earlier than with lower FC levels.20 Kallel et al., in another cohort study, showed that a level of FC ≥340 μg/g with the sensitivity of 80% and specificity of 91% could predict relapse in IBD patients,^21^ which is very close to the level achieved in our study(FC ≥341 μg/g). The levels achieved by Gisbert and Nakove were 150 μg/g and 250 μg/g, respectively; both lower than the levels achieved in our study.^22^,^23^ Retrospective design and lower sample size (below 100) may be the causes of the differences. In our results, the risk of relapse in younger patients at the time of diagnosis (≤42 years) was 9.24 times higher than in the older patients. Bitton et al. found that younger patients, especially those in the age group of 20-30 years, risked relapse earlier than other age groups.12 In a prospective study conducted in Seoul,^24^ the relapse rate in younger age groups was almost twice that of other age groups (69.2% vs. 32.4%; *P*=0.002).

The age obtained in this study is closed to the age level obtained from the present study to predict relapse. Adequate sample size and prospective design could be the reasons for the similarity of our results. The number of previous relapses is the other significant predictive factor of relapse in the present study. This factor was evaluated as a predictive factor of relapse in Indian patients, but there was no significant differences between relapsers and non-relapsers patients in that study.^25^ Bitton’s study,^12^ showed that the number of previous relapses has a significant relationship with relapse (hazard ratio: 1.14, *P*<0.001).

Major strengths of this study are its prospective design, large and adequate sample size, presentation of a novel risk-score formula for the prediction of flare in patients with UC. The two main limitations of our study were related to probable bias and financial restrictions. Some of the variables investigated in this study were dependent on the patient’s memory which could lead to recall bias. Therefore, as far as possible, patient records were used to avoid such bias. We sought to avoid inter-observer variation bias by using a general physician trained to fill out the questionnaire forms during the follow-up periods. Because of importance of clinical validation of each new risk scoring method, the authors recommended the use and evaluation of the results of the present study in clinical practices in the future studies.

## CONCLUSION

Four predictor variables were significant in the final model and were used in our risk-scoring formula. Our findings will assist physicians in the prediction of relapse risk. It is recommended that patients who achieve high scores are diligently observed, treated, and followed up.
